# Drug Resistance in Cancer: An Overview

**DOI:** 10.3390/cancers6031769

**Published:** 2014-09-05

**Authors:** Genevieve Housman, Shannon Byler, Sarah Heerboth, Karolina Lapinska, Mckenna Longacre, Nicole Snyder, Sibaji Sarkar

**Affiliations:** 1 School of Human Evolution and Social Change, Arizona State University, Tempe, AZ 85287, USA; E-Mail: ghousman@asu.edu; 2 Cancer Center, Department of Medicine, Boston University School of Medicine, Boston, MA 02118, USA; E-Mails: sbyler@bu.edu (S.B.); heerboth@bu.edu (S.H.); karolka@bu.edu (K.L.); nsnyder@bu.edu (N.S.); 3 Harvard Medical School, Boston, MA 02115, USA; E-Mail: Mckenna_Longacre@hms.harvard.edu

**Keywords:** cancer, drug resistance, epigenetics, methylation, cancer progenitor cells, combination therapy, review

## Abstract

Cancers have the ability to develop resistance to traditional therapies, and the increasing prevalence of these drug resistant cancers necessitates further research and treatment development. This paper outlines the current knowledge of mechanisms that promote or enable drug resistance, such as drug inactivation, drug target alteration, drug efflux, DNA damage repair, cell death inhibition, and the epithelial-mesenchymal transition, as well as how inherent tumor cell heterogeneity plays a role in drug resistance. It also describes the epigenetic modifications that can induce drug resistance and considers how such epigenetic factors may contribute to the development of cancer progenitor cells, which are not killed by conventional cancer therapies. Lastly, this review concludes with a discussion on the best treatment options for existing drug resistant cancers, ways to prevent the formation of drug resistant cancers and cancer progenitor cells, and future directions of study.

## 1. Introduction

**Figure 1:**
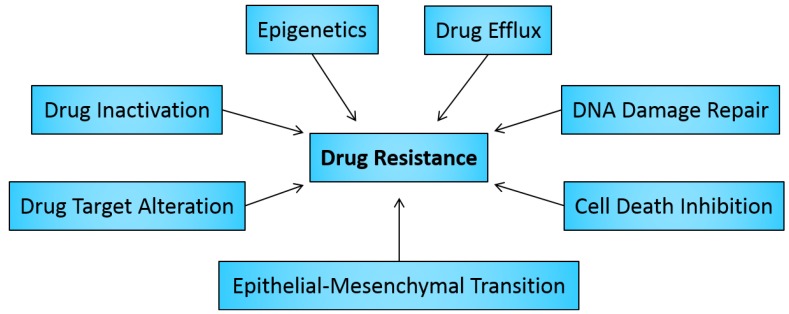
Categories of mechanisms that can enable or promote direct or indirect drug resistance in human cancer cells. These mechanisms can act independently or in combination and through various signal transduction pathways.

Drug resistance is a well-known phenomenon that results when diseases become tolerant to pharmaceutical treatments. This concept was first considered when bacteria became resistant to certain antibiotics, but since then similar mechanisms have been found to occur in other diseases, including cancer. Some methods of drug resistance are disease-specific, while others, such as drug efflux, which is observed in microbes and human drug-resistant cancers, are evolutionarily conserved. Although many types of cancers are initially susceptible to chemotherapy, over time they can develop resistance through these and other mechanisms, such as DNA mutations and metabolic changes that promote drug inhibition and degradation. In this review, we outline how drug resistance via drug inactivation, drug target alteration, drug efflux, DNA damage repair, cell death inhibition, and the epithelial-mesenchymal transition (EMT) develops in cancer in response to current treatments and how these problems are being addressed (Figure 1). We also consider how the cell heterogeneity inherent in cancerous tumors is involved in the development of drug resistance. Lastly, we conclude with a discussion on the emerging topic of epigenetics—how it contributes to drug resistance in cancer and its possible role in the development of cancer progenitor cells which are not killed by conventional cancer therapies.

## 2. Drug Resistance in Cancer

### 2.1. Drug Inactivation

Drug activation *in vivo* involves complex mechanisms in which substances interact with different proteins. These interactions can modify, partially degrade, or complex the drug with other molecules or proteins, ultimately leading to its activation. Many anticancer drugs must undergo metabolic activation in order to acquire clinical efficacy. However, cancer cells can also develop resistance to such treatments through decreased drug activation. One example of this is observed in the treatment of acute myelogenous leukemia with cytarabine (AraC), a nucleoside drug that is activated after multiple phosphorylation events that convert it to AraC-triphosphate [1,2]. Down-regulation or mutation in this pathway can produce a decrease in the activation of AraC, and this can lead to AraC drug resistance. Other important examples of drug activation and inactivation include the cytochrome P450 (CYP) system, glutathione-*S*-transferase (GST) superfamily, and uridine diphospho-glucuronosyltransferase (UGT) superfamily [3].

The CYP system is generally divided into two classes. Class I is composed of CYP1A1, CYP1A2, CYP2E1, and CYP3A4, which are well conserved, do not have important functional polymorphisms, and are active in the metabolism of drugs and procarcinogens. Class II is composed of CYP2B6, CYP2C9, CYP2C19, and CYP2D6, which are highly polymorphic and active in drug metabolism but not in procarcinogen metabolism [4]. Because class II gene sequences are more variable than those of class I, these CYP are better suited for drug metabolism and may have a role in the development of drug resistance in cancer. On the other hand, CYP1A1 and CYP1A2 metabolize procarcinogens into carcinogenic forms in the liver, and most anticancer drugs are metabolized by this method. Although CYP polymorphisms have not yet been associated with carcinogenesis, it is possible that mutations or alterations in CYP may change these proteins’ metabolic capabilities, such as increasing the breakdown of drugs and their secretion by the kidneys [5]. In this case, the drug would not maintain proper levels in the patient, and the cancer would therefore be considered resistant to it. The use of CYP and their suspected role in carcinogenesis has been well studied [3,6].

Many anticancer drugs require metabolic activation, and thus cancer cells can develop resistance through decreased drug activation. In patients with advanced ovarian cancer, treatment with platinum and taxane-based chemotherapy is applied post-operatively. One way resistance to platinum can occur is through drug inactivation by methallothionein and thiol glutathione, which activate the detoxification system [7]. Changes to apoptosis-related proteins can also result in drug resistance. For instance, apoptosis is promoted by the tumor suppressor protein p53 (*TP53*), in response to chemotherapy. *TP53* is mutated in 50% of cancers [8], and when mutation or deletion of this gene renders it non-functional, drug resistance can follow [9]. Alternatively, inactivation of P53 regulators, such as caspase-9 and its cofactor, apoptotic protease activating factor 1 (Apaf-1), can also lead to drug resistance [10].

Another important example of drug activation and inactivation is observed in the GST superfamily, which is a group of detoxifying enzymes that function to protect cellular macromolecules from electrophilic compounds. GSTs assist in the development of drug resistance through direct detoxification and by inhibiting the mitogen-activated protein kinase (MAPK) pathway [11]. Elevation of GST expression in cancer cells enhances detoxification of the anticancer drugs, which results in less efficient cytotoxic damage of the cells [12]. This increase is also associated with resistance to apoptosis initiated by a variety of stimuli [13].

Lastly, the UGT superfamily is a group of enzymes that catalyze glucuronidation. This process regulates the formation of inactive hydrophilic glucuronides with substrates such as steroids, bile acids, and xenobiotics including environmental carcinogens and cytotoxics. The *UGT1* and *UGT2* genes code 17 functional UGTs in humans, and these genes provide many tissues, such as the skin, breast, prostate gland, gut, and placenta, with a first line of metabolic defense from pathogenic substrates. However, widespread down-regulation of *UGT1A1* transcription and microsomal activity occurs in certain cancerous states [3]. The expression of *UGT1A1* is negatively regulated by DNA methylation at its promoter region, and irinotecan, a topoisomerase I inhibitor, is functional when this gene is silenced [14,15]. However, epigenetic changes that increase *UGT1A1* expression may enable resistance to irinotecan and other drugs. Overall, drug inactivation is a mechanism of cancer drug resistance that warrants further investigation.

### 2.2. Alteration of Drug Targets

A drug’s efficacy is influenced by its molecular target and alterations of this target, such as mutations or modifications of expression levels. In cancers, these types of target alterations can ultimately lead to drug resistance. For example, certain anticancer drugs target topoisomerase II, an enzyme that prevents DNA from becoming super- or under-coiled. The complex between DNA and topoisomerase II is usually transient, but these drugs stabilize it, leading to DNA damage, inhibition of DNA synthesis, and a halting of mitotic processes. Cancer cells can confer resistance in these circumstances through various means. Certain cell lines have become resistant to topoisomerase II-inhibiting drugs through mutations in the topoisomerase II gene [16,17,18]. Another type of anticancer drug targets signaling kinases, such as members of the epidermal growth factor receptor (EGFR) family and down-stream signaling partners such as Ras, Src, Raf, and MEK. Several of these kinases are constitutively active in certain cancers, and this promotes uncontrolled cell growth. In most circumstances, mutations cause the over-activation of these kinases; however, the same effect sometimes results from gene over-expression. Human epidermal growth factor receptor 2 (HER2), a receptor tyrosine kinase in the EGFR family, is overexpressed in 30% of breast cancer patients [14], and drug resistance can result after long term use of inhibitors targeting this kinase [19,20]. The increased response rates to EGFR inhibitors in certain lung cancers with EGFR tyrosine kinase domain mutations are reported with acquired resistance within one year. An *EGFR-T790M* gatekeeper mutation was reported in half of all cases [21,22]. Other genetic alterations such as chromosomal rearrangements and mutations in anaplastic lymphoma kinase are seen in anaplastic large-cell lymphoma [14,23]. Finally, resistance to paclitaxel and other taxanes has been observed in ovarian cancers via drug target alterations such as mutations in beta-tubulin, among other means [7].

Modified enzyme expression levels at drug target sites can also alter drug responses in cancer cells. For example, thymidylate synthase (TS) inhibitors, such as fluorouracil, ultimately inhibit the transcription of *TS* [14]. Fluorouracil becomes active when it is converted to fluorodeoxyuridine monophosphate (FdUMP), which forms a stable complex with TS and 5,10-methylenetetrahydrofolate (CH_2_THF). This TS-FdUMP-CH_2_THF complex results in a slowly reversible inactivation of the enzyme [24]. Another example of drug target alteration has been observed in the androgen receptor. In about 30% of prostate cancers, the androgen receptor is genomically amplified, which enables these cancers to become resistant to androgen deprivation therapy with the drugs leuprolide and bicalutamide [14,25]. These drugs cannot inhibit all the molecular targets present, and thus these cancers are considered resistant to them.

In addition to the changes in specific drug targets, drug resistance is also achieved by alteration in the signal transduction process that mediates drug activation. For example, the treatment of HER2-positive breast cancer tumors with trastuzumab (Herceptin), a humanized monocolonal antibody, has had high levels of efficacy in combination with chemotherapy. However, many patients who initially respond to trastuzumab develop resistance and relapse, despite continued treatment. Trastuzumab also has limited efficacy as a single agent, and some patients do not respond to treatment at all, despite being HER2-positive. The mechanism of resistance is thought to be associated with cell cycle inhibition, co-expression of growth factor receptors, activation of PI3K/Akt pathway, and loss of phosphatase and tensin homolog (PTEN) function [26,27]. Insulin-like growth factor 1 receptor (IGF1R) levels have been found to significantly increase in the trastuzumab-resistant cell line as compared to the non-resistant parental cell line. These results confirm that IGF1R inhibition improves response to trastuzumab in HER2-positive breast cancer cells, and suggest that dual targeting of IGF1R and HER2 may improve response in HER2-positive tumors [28]. Others have also shown that activation of the PI3K/Akt pathway through *PI3KCA* mutations, PTEN loss, or both is associated with accelerated disease progression and decreased survival, indicating the adverse effect of this pathway’s status on trastuzumab efficacy [29].

In the case of chronic myeloid leukemia (CML), break point cluster-Abelson (BCR-ABL) tyrosine kinase is generated from the chromosomal translocation t(9;22). Imatinib is a tyrosine kinase inhibitor that specifically targets the BCR-ABL protein and induces remission in patients with CML. Unfortunately, the majority of CML patients treated with imatinib develop resistance at some point during therapy. Some patients may fail to respond to initial treatment with imatinib (primary resistance), while others stop responding with prolonged therapy after an initial response (acquired resistance). Several mechanisms of imatinib resistance have been proposed that account for loss of imatinib efficacy in patients with CML. Imatinib resistance can be caused by point mutations in the *ABL* geneand amplification of the *BCR-ABL* fusion gene [30]. In addition to these BCR-ABL-dependent mechanisms, BCR-ABL-independent mechanisms of imatinib resistance have been proposed, which involve drug transporter and signaling cascades. Investigation of *SOCS-3* gene methylation and downstream effects in BCR-ABL-positive CML cells resistant to imatinib found that this epigenetic effect resulted in STAT3 protein activation that led to uncontrolled cell proliferation [31]. Others proposed that over-expression of the efflux drug transporter P-glycoprotein (Pgp) partially contributed to imatinib resistance in imatinib-resistant K562 CML cells having no *BCR-ABL* mutations [32]. Additionally, researchers have determined that the BCR-ABL-independent activation of ERK1/2 contributes to imatinib resistance in K562/R cells and that ERK1/2 could be targeted for treatment in CML patients with imatinib resistance due to this mechanism [33].

Another example of alterations in signaling mechanisms is tamoxifen resistance in breast cancer. Tamoxifen acts as an estrogen receptor (ER) antagonist. However, ER signaling has a complex interaction with other growth signaling pathways in breast cancer cells, thus enabling drug resistance through various mechanisms. For example, in tumors with active growth factor receptor signaling (e.g., *HER2* amplification), tamoxifen may lose its estrogen antagonist activity and acquire more agonist-like activity, resulting in tumor growth stimulation [34]. Additionally, expression of EGFR and HER2, which are barely detected in control estrogen-treated tumors, was found to increase slightly with tamoxifen and markedly increase when tumors became resistant [35]. Understanding this and other methods of drug target alteration is important for diagnosing and developing new therapies to treat drug-resistant cancers.

### 2.3. Drug Efflux

One of the most studied mechanisms of cancer drug resistance involves reducing drug accumulation by enhancing efflux. Members of the ATP-binding cassette (ABC) transporter family proteins enable this efflux and are important, well-studied regulators at the plasma membranes of healthy cells. ABC transporters are transmembrane proteins present not only in human cells, but in all extant phyla, functioning to transport a variety of substances across cellular membranes. Though a transporter’s structure varies from protein to protein (e.g., there are 49 known members of the ABC family in humans), they are all classified by the presence of two distinct domains—a highly conserved nucleotide binding domain and a more variable transmembrane domain. [36] When a given substrate binds to the transmembrane domain, ATP hydrolysis at the nucleotide binding site drives a change in conformation that pushes the substrate out of the cell. This efflux mechanism plays an important role in preventing over accumulation of toxins within the cell [37]. Not surprisingly, ABC transporters are highly expressed in the epithelium of the liver and intestine, where the proteins protect the body by pumping drugs and other harmful molecules into the bile duct and intestinal lumen. They also play a large role in maintaining the blood-brain barrier [38,39].

While efflux via ABC transporters is a normal physiological process, it is also a known mechanism of drug resistance in cancer cells. Three transporters—multidrug resistance protein 1 (MDR1), multidrug resistance-associated protein 1 (MRP1), and breast cancer resistance protein (BCRP)—are implicated in many drug resistant cancers. All three transporters have broad substrate specificity and are able to efflux many xenobiotics, including vinca alkaloids, epipodophyllotoxins, anthracyclines, taxanes, and kinase inhibitors, from cells. Thus, they protect cancer cells from many first line chemotherapies. *MDR1*, which produces Pgp, was the first of these to be identified and has been studied extensively [40,41,42]. Normal expression of the *MDR1* gene in the colon, liver, and kidney is increased when these tissues become cancerous. Interestingly, in one study it was shown that treatment with doxorubicin induced a large increase in *MDR1* expression in lung cancer cells, while no significant change in expression was observed in normal lung cells [43], suggesting that there are both intrinsic and acquired mechanisms of *MDR1* over-expression. Tissues that do not normally express *MDR1*, such as lung, breast, and prostate cells, are often drug resistant due to the expression of the related transporters MRP1 or BCRP. BCRP protects normal cells from the effects of toxins like xenobiotics, maintains heme and folate homeostasis, and is expressed in stem cells. Many studies in various types of cancer have shown that increased expression of either of these transporters in tumor cells confers poor clinical outcomes. In one study of neuroblastoma, it was found that high levels of *MRP1* expression were significantly correlated with poor clinical outcomes [44]. Similarly, expression of *BCRP* was predictive of drug response and survival rates in small cell lung cancer patients. It is sometimes possible to decrease drug efflux with the use of a BCRP inhibitory drug, such as Gefitinib. This particular drug is a tyrosine kinase inhibitor that functions to block the transporter function of BCRP, reversing drug resistance [45]. While few compounds have been identified to directly inhibit BCRP, it is clear that estrogen plays a large role in regulating its expression. One study showed that 17b estradiol down-regulates the expression of *BCRP* in breast cancer cells, thereby increasing the concentration of chemotherapeutic drugs in the cancer cells [46]. Overall, inhibition of these transcripts may help to sensitize cancer cells to drug treatments.

As mentioned previously, the constitutive activation of signaling molecules like kinases drives the cell cycle out of control and results in cancer. Additionally, these proteins also regulate Pgp expression and can thereby modulate the environment to enable the development of drug resistance. Estrogen down-regulates the protein synthesis of Pgp in ER-positive breast cancer cells but not in ER-negative breast cancer cells or doxorubicine resistant ER-negative ovarian cancer cells [47,48]. Conversely, over-expression of proteins involved in the MAPK pathway, such HRas, c-Raf, MEK1/2, ERK1/2, which act downstream of receptor tyrosine kinases, increases the expression of Pgp. While inhibitors of the extracellular signal-regulated kinases (ERK) pathway down-regulate Pgp expression, growth factors like EGF and FGF increase it [49]. Interestingly, inhibition of HSP90, a chaperone protein that stabilizes many signaling proteins, also down-regulates Pgp [50]. Overall, these results suggest that Pgp expression and stability are tightly regulated and advantageous to tumor cell progression. Targeting these oncogenic kinases that are often activated in cancers may be useful in reducing Pgp expression and sensitizing cancer cells to other drugs.

### 2.4. DNA Damage Repair

The repair of damaged DNA has a clear role in anticancer drug resistance. In response to chemotherapy drugs that either directly or indirectly damage DNA, DNA damage response (DDR) mechanisms can reverse the drug-induced damage. For example, platinum-containing chemotherapy drugs such as Cisplatin cause harmful DNA crosslinks, which can lead to apoptosis. However, resistance to platinum-based drugs often arises due to nucleotide excision repair and homologous recombination, the primary DNA repair mechanisms involved in reversing platinum damage [51,52,53]. Thus, the efficacy of DNA-damaging cytotoxic drugs depends on the failure of the cancer cell’s DDR mechanisms. Inhibition of repair pathways used in conjunction with DNA damaging chemotherapy could sensitize cancer cells and therefore increase efficacy of the therapy.

The therapeutic potential of targeting DDR mechanisms is especially exciting due to the prevalent dependence of cancers on a compensatory repair mechanism. Dysregulation or impairment of certain DDR genes and mechanisms either by mutations or epigenetic silencing are common in many cancers [54,55,56]. However, other DDR mechanisms can be up-regulated to compensate for the dysfunctional pathways. Although increased DNA repair activity can lead to increased resistance, this compensation also provides two opportunities for chemotherapy. First, targeting the overactive DDR pathway with chemotherapeutic drugs could leave cancers especially vulnerable to DNA-damaging drugs. Alternatively, knowledge of the dysfunctional DDR could allow proper prescription of a DNA-damage causing drug, which induces damage only repaired by the defective pathway. In both chemotherapy strategies, it is essential to identify the over-active and under-active DDR mechanisms.

The DNA repair via O6-methylguanine DNA methyltransferase (MGMT) illustrates many of the challenges and promises of targeting DDR pathways for anticancer therapy. Some chemotherapy drugs induce guanine O6 alkylation. MGMT repairs such an alkylated nucleotide, converting it back to guanine before mismatch can occur. Over-expression of *MGMT* has been shown to protect hematopoietic stem cells from alkylating agents [57]. However, many tumors also have high MGMT levels [58], yielding them resistant to alkylating agents. Inhibiting this DDR mechanism could therefore prevent resistance and make cancer cells more vulnerable to alkylating agents.

Although drugs targeting MGMT have been developed, few have shown much promise, and none are FDA approved [54]. In addition to only marginal clinical efficacy, some of these drugs also show toxicity due to a lack of specificity for cancer cells. Accordingly, drugs currently in trial such as O6-benzylguanine sensitize healthy cells to cytotoxic drugs [54,59,60]. One possible way to avoid this problem could be to individualize chemotherapy by identifying *MGMT* promoter CpG methylation as a biomarker for increased sensitivity to O6-guanine alkylating agents. *MGMT* promoter methylation is often clinically associated with uncertain prognosis due to the genomic instability caused by silencing a DDR mechanism. However, studies also show that many glioma patients with epigenetically silenced *MGMT* genes have increased disease-free and overall survival rates [61]. The role of methylation in regulating *MGMT* is further discussed in the epigenetics section.

### 2.5. Cell Death Inhibition

Cell death by apoptosis and autophagy are two important regulatory events. Although these processes are antagonistic to one another, they both contribute to cell death. Apoptosis has two established pathways: an intrinsic pathway mediated by the mitochondria that involves B-cell lymphoma 2 (BCL-2) family proteins, caspase-9 and Akt, and an extrinsic pathway that involves death receptors on the cell surface. The intrinsic and extrinsic pathways merge through the activation of down-stream caspase-3, which ultimately causes apoptosis. However, there is also additional cross-talk between the pathways.

In several types of cancers, BCL-2 family proteins, Akt, and other antiapototic proteins are highly expressed and down-stream transcription modulators like NF-κB and STAT are highly active, making these good targets for drug development. Recombinant forms of tumor necrosis factor related apoptosis-inducing ligand (TRAIL) and agonistic antibodies to these receptors can induce apoptosis through the activation of caspase-8. Clinical trials with TRAIL failed to produce significant results, but TRAIL in combination with other cytotoxic drugs is showing promise [62,63]. Several other drugs, including BCL-2 family inhibitors, histone deacetylase inhibitors (HDACi), protease inhibitors, and kinase inhibitors, are also showing promise in recent drug trials [14,63,64,65,66]. In fact, many new BCL-2 family protein inhibitors are effective in inducing apoptosis in cancer cells, but prolonged use can produce resistance. Additionally, it has been shown that HDACi sensitize breast cancer cells to TRAIL in a mouse model [63] and to a protease calpain inhibitor in cell cultures [64]. Moreover, in two different studies it was shown that HDACi sensitize ovarian cancer cells to the telomere analog GT-oligo, and GT-oligo sensitizes ovarian cancer cells to TRAIL [65,66]. Many cancer drugs also induce apoptosis via the activation of c-Jun N-terminal kinases (JNK), which is downstream of the MAPK pathway. TRAIL induces apoptosis through JNK activation [65,66], and inhibition of the JNK signaling pathway leads to a decrease in cisplatin-induced apoptosis. All of these results suggest that cancer cells, including those which are drug resistant, can be effectively treated by using one drug that makes the cells susceptible to death through the altered expression or regulation of cell death pathway members in combination with another cytotoxic drug that kills the cells in their vulnerable states. HDACi are epigenetic drugs, and the implications of using these types of drugs as synergistic agents to sensitize normal and drug-resistant cancer cells is discussed further in the epigenetic section of this review.

Autophagy is caused by phagolysosomal death in an acidic lysosomal pH. Drugs such as chloroquine and its derivatives prevent this process by raising the pH to inactivate digestive enzymes in lysosomes. These drugs have primarily been used in the treatment of malaria, but they have also been shown to be beneficial in sensitizing cancer cells to other drugs. For instance, fluorouracil in combination with chloroquine is more effective at treating cancer cells than fluorouracil alone [67]. Additionally, hydroxychloroquine, a derivative of chloroquine, has been shown to inhibit autophagy in cancer cells and restore sensitivity to ER pathway inhibitors, such as tamoxifen, in ER-positive cancer cells [68]. Overall, chloroquine is thought to play a role in inhibiting autophagy-dependent resistance to chemotherapy [67], which makes it especially important in the field of drug-resistant cancers. These examples and the roles of apoptosis and autophagy in cancer drug resistance have been extensively discussed elsewhere [14].

### 2.6. Epithelial-Mesenchymal Transition and Metastasis

The epithelial to mesenchymal transition (EMT) is a mechanism by which solid tumors become metastatic. Metastasis itself is a complex phenomenon that includes changes in a cancer cell and the stromal cells that make up its environment. It also includes angiogenesis, which is the formation of new blood vessels around metastatic tumors. During EMT, cells within a tumor reduce the expression of cell adhesion receptors, including integrins and cadherins, which help in cell-cell attachment, and increase the expression of cell adhesion receptors that induce cell motility. Cell motility is also dependent on cytokines and chemokines, which may be released by cells in the microenvironment of tumors or by the tumors themselves. Additionally, higher expression of metalloproteases on the surface of tumors helps to clear the road for the cells to move outward, promoting metastasis. The role of EMT in cancer drug resistance is an emerging area of research [69,70].

Recent articles point toward the involvement of cancer progenitor cells, which are sometimes referred to as cancer stem cells, in the formation of metastatic cancer cells, and this might explain why cancer can relapse at distant sites after apparently successful treatment and remission [71,72,73,74,75]. Death of these cancer progenitor cells via epigenetic drug treatment may be one way to prevent remote site metastasis. Several factors during EMT play significant roles in the development of drug resistance, but these are dependent on the metastatic grade of the tumor, which is defined as the level of differentiation and degree of EMT. For example, in ERBB2 (HER2) positive breast cancer, tumors that express high levels of β1 integrins develop more resistance to antibody inhibitors such as transtuzumab [76]. This finding reaffirms previous studies that found that the ligation of β1 integrins protects leukemia cells from drug induced cell death [77]. Additionally, integrin receptors and receptor tyrosine kinases need to associate in order for breast cancer to progress [78,79].

**Figure 2:**
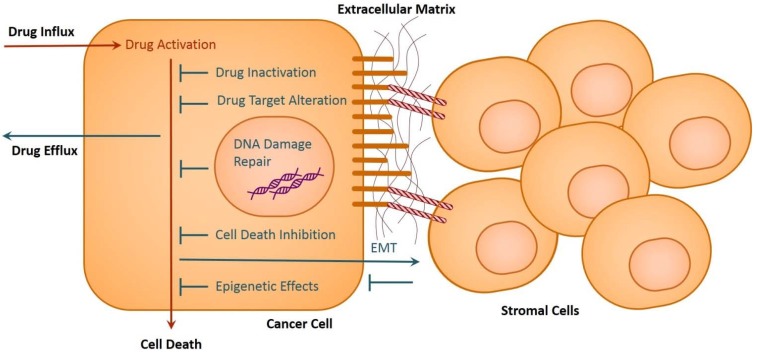
Depiction of the primary mechanisms that enable cancer cells to become drug resistant. These include drug inactivation, alteration of drug targets, drug efflux, DNA damage repair, inhibition of cell death, EMT, and epigenetic effects. In the case of EMT, stromal cells assist in this process and signal for improved drug resistance in cancer cells. Cell adhesion molecules on stromal cells and extracellular matrix proteins attach to the cell adhesion molecules on cancer cells. Stromal cells and cancer cells also secrete factors that regulate EMT. The depiction displays a simplified example of these cell interactions.

Drug resistance in cancer cells may also develop during the signaling processes of differentiation, which are essential for EMT. For example, the increased expression of integrin αvβ1 in colon cancer positively regulates transforming growth factor β (TGFβ) expression, which is required for EMT, and it further serves as a survival signal for cancer cells against drugs [80]. Integrin αvβ1 interacts with stromal cell adhesion molecules to convey such signals [80]. Similarly, β3 integrin and src regulate TGFβ mediated EMT in mammary cancer [81]. Ligation of integrin β1 provides proliferative and survival signal-mediated FAK kinase in lung cancers [82]. Autocrine signaling provided by vascular endothelial growth factor (VEGF) and Flt-1 help the cancer cell survival process [80]. Selectin and other cell adhesion receptors, which interact with the extracellular matrix and cell adhesion receptors of stromal cells, also participate in the process of EMT and cell survival [83,84,85,86,87] (Figure 2). The differentiation process during EMT generates more metastatic cancer cells with different cellular morphology, which needs cytoskeletal re-arrangement [88]. Recent studies suggest a possible connection of actin binding protein L-plastin in colon cancer progression and prognosis [89]. T-plastin is implicated in the progression of lymphomas and their drug resistance [90].

The role of stromal cells in causing drug resistance has also been investigated. B-Raf is an intermediate kinase in the down-stream signaling pathway initiated by receptor tyrosine kinases. Resistance against B-Raf inhibitor drugs was observed in melanoma cells when they were co-cultured with fibroblast cells [91], indicating that stromal cells may influence the development of drug resistance. This is one reason why so many drugs fail clinical trials in *in vivo* animal models despite high efficacy against cancer in cell cultures.

EMT and cancer metastasis involve numerous variables. Metastatic cancer cells are often a heterogeneous population, in which cell differentiation is not uniform. This difference is one of the reasons why some patients are more responsive to treatments than the other. It appears that EMT, while favoring the formation of more metastatic cancer cells, also provide signals for increasing survival which may cause drug resistance in some if not all the cells present in a tumor. Since this is a very complex and dynamic mechanism, thorough investigation is necessary to fully understand each step in the regulation of drug action and thus, drug resistance.

### 2.7. Cancer Cell Heterogeneity

In addition to the development of drug resistance in cancer progenitor cells and adult cancer cells by the mechanisms previously discussed, another aspect of cancer relapse is the enrichment of drug resistant cancer cells already present in the heterogeneous cancer cell population. Recent studies show that a fraction of cells within this heterogeneous population have stem cell properties and are usually drug resistant. In addition, another small fraction of adult cancer cells also possess drug resistance capabilities. The treatment of cancers, by definition, kills only drug sensitive cancer cells, and thus the drug resistance cancer cells survive and can expand and contribute to pathology over time. Some of these resistant cancers cells may be in the circulation and can form tumors in distant organs. However, heterogeneity is observed in cancer cells both in circulation and in solid tumors.

A recent study on acute myeloid leukemia determined two coexisting dominant clones. One was drug sensitive and the other drug resistant. It is possible that re-occurrence of this disease in patients after successful therapy may be the result of cancer cell growth from the drug resistant clone [92]. This possibility exists in all forms of cancer, as all tumors are heterogeneous, due to aberrant DNA repair mechanisms and cell death pathway dysregulation. A clonal composition study of breast cancer revealed that breast cancers may have monogenomic or multiple genomic tumors [93]. Polygenomic tumors contain many different types of clonal subpopulations, all of which may have different drug sensitivities and resistance characteristics [93].

An analysis of pancreatic cancer samples showed that tumor progression involves telomere dysfunction and cell cycle deregulation and that these changes occur in the early stage of carcinogenesis [94]. However, the metastatic process is not as well defined, and with heterogeneity as an outcome, the possibility of different drug sensitivities and drug resistance characteristics among clonal subpopulations arises [94]. Taken together, the drug resistance of cancer progenitor cells and the acquired drug resistance of cancer cells following EMT or other mechanisms pose a very complex challenge for the development of better therapies to reduce the relapse of cancers.

## 3. Role of Epigenetics in Cancer Drug Resistance

An important set of mechanisms that cause resistance to cancer treatment and that have not been readily discussed are epigenetic modifications, which can also influence carcinogenesis. The two main types of epigenetic changes are DNA methylation and histone modification via acetylation or methylation. DNA methylation consists of methyl groups binding to cytosines at CG-dinucleotides within regions known as CpG islands, primarily found in upstream gene promoter regions. However, methylation can occur at other loci throughout the genome. Conversely, histone modifications alter chromatin conformation. For example, histone acetylation opens the chromatin, while deacetylation closes it. These mechanisms ultimately regulate the expression of genes throughout the chromosome, and in cancer, this normal regulation is broken. For example, tumor suppressor genes are often silenced via hypermethylation, and oncogenes are over-expressed via hypomethylation. However, epigenetic mechanisms are usually reversible, and researchers may be able to take advantage of this opportunity to develop treatments that can counteract drug resistant cancers.

This review initially focused on how established mechanisms cause resistance in cancer cells. Interestingly, many of these well-studied mechanisms may also be influenced by epigenetic changes. More recent studies suggest that epigenetic alterations, such as histone methylation and acetylation, may play a role in the development of drug resistance. One study proposed that hypermethylation of the *MDR1* promoter is associated with transcriptional repression and chromatin structural changes [95]. Others have also suggested that DNA methylation is associated with acquired multidrug resistance. In experiments expanding on this idea, demethylation of the *MDR1* promoter in cancer cell lines was found to be strongly associated with the acquisition of a multidrug resistant phenotype [96]. Overall, methylation at this promoter controls *MDR1* transcription, increases drug resistance, and decreases drug accumulation, making it an excellent target for epigenetic treatment. Specifically, anti-methylation drugs might be useful in sensitizing multidrug resistant cancer cells to other types of drugs.

Epigenetic mechanisms can also influence DNA damage repair. For example, DNA mismatch repair processes can be lost due to hypermethylation of the human mutL homolog 1 (hMLH1) gene promoter, and this can lead to cancer development. In one study, tumor-bearing mice were treated with nontoxic doses of the demethylating agent 2-deoxy-5-azacytidine (DAC). While the re-expression of *hMLH1* is associated with a decrease in *hMLH1* promoter methylation, the DAC treatment was not found to have an effect on the rate of tumor growth. However, it did sensitize the tumors to other drugs, including cisplatin, carboplatin, temozolomide, and epirubicin [97]. DAC may have a role in increasing the efficacy of chemotherapy for patients with tumors characterized by high *hMLH1* promoter methylation and low hMLH1 expression. Similarly, another experiment showed that demethylation of the *hMLH1* promoter by DAC restores mismatch repair proficiency and drug sensitivity to 5-fluorouracil in colorectal cancer cells [98].

The DNA repair enzyme MGMT inhibits the killing of tumor cells by alkylating chemotherapy agents. Methylation of *MGMT* causes gene silencing and decreased MGMT production. Epigenetic alteration of *MGMT* expression has been associated with a modified chromatin configuration. Cells can acquire resistance to N-methyl-N-nitrosurea, a methylating chemotherapy agent, by either reactivating a previously silenced *MGMT* gene, or by repressing the *hMSH6* mismatch repair gene. The number of active MGMT molecules at the time of methylation determines the capacity of a cell for MGMT repair. Treatment with chemical methylating agents alters gene expression patterns by increasing genomic DNA methylation, which ultimately leads to increased repair or tolerance of O6-methylguanine and the emergence of chemotherapy resistance [99]. Other researchers have studied gliomas to determine whether *MGMT* promoter methylation is related to the responsiveness of a tumor to alkylating agents, and found that this methylation was associated with tumor regression and prolonged survival rates [100].

Human breast cancer cells can also exhibit drug resistance via epigenetic mechanisms. For example, methotrexate resistance in MDA-MB-231 breast cancer cells is caused by an inherent defect in drug uptake and a lack of reduced folate carrier (RFC) expression. In one study, the treatment of MDA-MB-231 cells with the DNA methylation inhibitor DAC improved methotrexate uptake but also restored RFC expression, which promoted methotrexate efflux. These results suggest that DAC counteracts some methotrexate-resistance mechanisms while improving others [101]. In another study, an inverse relationship was found between tamoxifen resistance and methylation of the *ERβ* gene. In general, tamoxifen-resistant tumors showed denser *ERβ* gene methylation than control tumors [102].

Epigenetically mediated forms of drug resistance are also observed in other cancers. For instance, melanoma cells, which are notoriously unresponsive to chemotherapy, can acquire resistance to the chloroethylating drug fotemustine. One study determined that this acquired resistance is associated with high MGMT activity and that the *MGMT* gene in fotemustine resistant cells was hypermethylated. However, these cells were effectively sensitized when treated with DAC [103]. Additionally, some prostate cancers exhibit androgen resistance that may be due to transcriptional inactivation of the androgen receptor gene caused by DNA methylation. Cytosine DNA methyltransferase inhibitors have been found to restore androgen responsiveness in androgen-refractory tumor cells, though, and these cells are then responsive to growth inhibition by anti-androgens [104]. Overall, epigenetic alterations have been increasingly recognized as a cause of drug resistance in many different kinds of cancer. Thus, epigenetic therapy could be utilized as a priming therapy to sensitize drug-resistant cancer cells in conjunction with conventional and targeted chemotherapy.

In addition to the development of drug resistance, epigenetics plays a significant role in cancer progenitor cell (or cancer stem cell) formation and cancer progression [73,74]. Cancer progenitor cells are not killed by conventional cancer therapies and are a major cause of cancer relapse. Addressing this problem could reduce relapse as well as provide a means by which to handle drug resistant cancer cells. Thus, this is an important topic to consider. Cancer progenitor cell formation is a complex process. The current paradigm suggests that a combination of environmental and genetic changes, such as random mutations, increased signaling processes, stromal influences, hormonal imbalances, and germ-line mutations make adult and stem cells susceptible to progenitor cell formation. However, it is reasonable to suspect that a common trigger ignites the progression of these susceptible cells, and we have proposed that epigenetic alterations may serve as this common trigger to stimulate the development of normal cells with a cancer predisposition into cancer progenitor cells [73,74]. For example, MDR1 expression increases in early cancer progenitor cells of the myeloid lineage. Over-expression of *MDR1* was also found to be associated with the expression of CD34 antigen, a marker for progenitor cells of this lineage. Interestingly, this correlation was observed in myelodysplasias and myeloblast leukemia [16]. Cancer cells are opportunistic in silencing tumor suppressor genes by methylation, increasing expression of telomeres by methylation, and enhancing the expression of oncogenes by hypomethylation. These are the characteristics which may drive predisposed stem cells to form cancer progenitor cells. This idea is partially supported by the fact that cancer progenitor cells are usually drug resistant. Higher expression of the *MDR1* gene could be one mechanism by which they acquire drug resistance. In contrast, mature leukemia cells are drug sensitive and show low levels of *MDR1* expression. However, mature leukemia cells may have a population of cancer progenitor cells that highly express *MDR1*, and this *MDR1* expression again increases when these cells become drug resistant. As discussed, epigenetics can regulate the expression of the *MDR1* gene, and this reversible epigenetic mechanism could be a prime target for drug therapies.

Reversing the epigenetic changes that assist in cancer progenitor cell formation should effectively kill these cells and should consequently stop tumor growth and decrease the chance of relapse. Tumorigenesis requires metastatic potential, and cell differentiation is essential for the stage-specific formation of increasingly metastatic tumor forms or grades. Since cell growth must slow down before a cell differentiates, we have proposed that epigenetic switches, which can simultaneously enhance differentiation and repress growth, regulate the stage-specific development of more metastatic cancer [73,74,75]. Therefore, epigenetic modifications may also play a key role in tumor formation and tumor metastasis, further making them ideal targets for therapy in the context of drug resistance.

Theoretically, a combination of epigenetic drugs with conventional chemotherapy should be more effective in treating tumors and drug resistant cancers. Several recent studies have shown encouraging results to support this hypothesis [73,74,75]. One study has shown that HDACi treatment demethylates and re-expresses tumor suppressor genes [105], leading to the sensitization of cancer cells to other cytotoxic drugs. Additionally, HDACi in combination with the calpain protease inhibitor calpeptin has been shown to enhance growth inhibition of breast and ovarian cancer cells [64,106]. Furthermore, the combination of HDACi and TRAIL in mouse models was found to reduce tumor size by inducing apoptosis [37], and the combination of HDACi and GT-oligo increases ovarian cancer cell death [65]. We have proposed that demethylation and re-expression of tumor suppressor genes render cancer cells susceptible to other cytotoxic drugs [73,74,75,107]. Drug resistant cancer cells may be similarly sensitized by demethylation to other cytotoxic agents as well. Recent clinical studies suggest that pretreatment with epigenetic drugs can reduce cancer relapse and be more effective for treating drug resistant cancers [108]. For example, one study determined that lung cancer patients who were treated with the epigenetic drugs DAC and HDACi prior to conventional chemotherapy had lower incidences of relapse [108]. Two other studies demonstrated that MAPK pathway inhibitors in combination with HDACi suppressed cAMP mediated resistance in melanoma cells [109] and that pre-treatment of platinum drug resistant ovarian cancer cells with HDACi and methylation inhibitors sensitized these cells to cisplatin-mediated cell death [110]. In this last study, epigenetic drug treatment resulted in the re-expression of *RGS10*, an important regulator of cell survival and chemoresistance in ovarian cancer. Hypermethylation and histone deacetylation silences this gene in drug resistant ovarian cancer cells, and re-expression of this gene made these cells susceptible to platinum drugs. Overall, these results indicate that pretreatment using epigenetic drugs in combination with conventional therapies may be beneficial for reducing cancer relapse and improving drug resistant cancer treatment. The role of epigenetic drugs in treating a myriad of diseases has been discussed extensively elsewhere [111,112] and is an important area of research to be further pursued.

## 4. Conclusions

Cancer drug resistance is a complex phenomenon that is influenced by drug inactivation, drug target alteration, drug efflux, DNA damage repair, cell death inhibition, EMT, inherent cell heterogeneity, epigenetic effects, or any combination of these mechanisms. The current paradigm states that combination therapy should be the best treatment option because it should prevent the development of drug resistance and be more effective than any one drug on its own [73,74,75,107,111,112]. Therefore, such treatment regimens should be considered and developed to counteract the increasing prevalence of drug resistance in cancers. Cancer progenitor cells are often drug resistant as well. These progenitor cells can persist in patients seemingly in remission, and they are able to remain stationary or migrate to other sites during metastasis. Thus, cancer progenitor cells can cause cancer relapse at the original tumor site or in distant organs. The next step in anticancer therapy development should target the elimination of such cancer progenitor cells. Additionally, the existence of a small population of drug resistant cancer cells poses another complexity that is difficult to address [92,93,94]. These drug resistant cancer cells also contribute to cancer relapse after apparent remission. It will be interesting to determine how much contribution cancer progenitor cells or drug resistant cancer cells render to generate drug resistance. Therefore, it is important to continue efforts to understand the underlying mechanisms of cancer drug resistance and to identify therapies that can treat cancers no longer susceptible to current treatments. Epigenetic drugs may assist in this endeavor as they are thought to be capable of sensitizing drug resistant cancer cells to other drugs [73,74,75,107,111,112], and recent studies have supported these propositions [108,110]. Further research in this direction is needed to improve overall understanding and treatment of drug resistant cancers.

## Abbreviations

EMT: epithelial-mesenchymal transition
AraC: cytarabine
CYP: cytochrome p450
GST: glutathione-S-transferase
UGT: uridine diphospho-glucuronosyltransferase
TP53: tumor protein p53
Apaf-1: apoptotic protease activating factor 1
MAPK: mitogen-activated protein kinase
EGFR: epidermal growth factor receptor
HER2: human epidermal growth factor receptor 2
TS: thymidylate synthase
FdUMP: fluorodeoxyuridine monophosphate
CH_2_THF: 5,10-methylenetetrahydrofolate
PTEN: phosphatase and tensin homolog
IGF1R: insulin-like growth factor 1 receptor
CML: chronic myeloid leukemia
BCR-ABL: break point cluster-Abelson
Pgp: P-glycoprotein
ER: estrogen receptor
ABC: ATP-binding cassette
MDR1: multidrug resistance protein 1
MRP1: multidrug resistance-associated protein 1
BCRP: breast cancer resistance protein
ERK: extracellular signal-regulated kinases
DDR: DNA damage response
MGMT: O6-methylguanine DNA methyltransferase
BCL-2: B-cell lymphoma 2
TRAIL: tumor necrosis factor related apoptosis-inducing ligand
HDACi: histone deacetylase inhibitors
hMLH1: human mutL homolog 1
DAC: 2'-deoxy-5-azacytiding
RFC: reduced folate carrier
